# Supplementary material for: Political context and immigrants’ work-related performance errors: Insights from the National Basketball Association
Source: PLoS One. 2023 Nov 1;18(11):e0289019. doi: 10.1371/journal.pone.0289019 (PMC10619861; doi:10.1371/journal.pone.0289019)
Supplement: Attachment — Submitted filename: Response to Reviewers.docx [file pone.0289019.s001.docx]

Dear Dr. Joseph Donlan,

Thank you for giving us the opportunity to revise and resubmit our manuscript. We are grateful that you and the Reviewer have provided us with valuable insight on how to strengthen our manuscript and bring it in line with PLOS ONE’s formatting requirements. Below we discuss your comments in detail, with each of our responses presented in blue.

Editor Comments to the Author

1. Please ensure that your manuscript meets PLOS ONE's style requirements, including those for file naming. The PLOS ONE style templates can be found at

https://journals.plos.org/plosone/s/file?id=wjVg/PLOSOne_formatting_sample_main_body.pdf and

https://journals.plos.org/plosone/s/file?id=ba62/PLOSOne_formatting_sample_title_authors_affiliations.pdf

Thank you for referring us to these style templates. We have now gone through and formatted our manuscript and title page according to PLOS ONE’s style requirements.

2. You indicated that ethical approval was not necessary for your study. We understand that the framework for ethical oversight requirements for studies of this type may differ depending on the setting and we would appreciate some further clarification regarding your research. Could you please provide further details on why your study is exempt from the need for approval and confirmation from your institutional review board or research ethics committee (e.g., in the form of a letter or email correspondence) that ethics review was not necessary for this study? Please include a copy of the correspondence as an ""Other"" file.

Thank you for bringing this to our attention. Our study was exempt from review due to its non-invasive nature and reliance on publicly available data. Confirmation of exemption from our university’s institutional review board (in the form of e-mail correspondence) has been included in our submitted revision documents as an “Other” file. Please let us know if additional information is required.

3. Please review your reference list to ensure that it is complete and correct. If you have cited papers that have been retracted, please include the rationale for doing so in the manuscript text, or remove these references and replace them with relevant current references. Any changes to the reference list should be mentioned in the rebuttal letter that accompanies your revised manuscript. If you need to cite a retracted article, indicate the article’s retracted status in the References list and also include a citation and full reference for the retraction notice.

Thank you for reminding us to ensure that our reference list is complete and correct. We have gone through our reference list and confirmed its accuracy. Furthermore, we have included additional references in our revised manuscript to support our arguments. These changes are visible in the revised manuscript with highlighted track changes.

Reviewer #1 Comments to the Author

The research conducted is both original and rigorous, and it addresses an important current issue. The variables are well-defined and the use of objective field data is a strength. However, my main concern is that the authors do not delve deeply into the impact on native players. Do the authors believe that the right-wing anti-immigration discourse is responsible for both expected effects, as described in H1 and H2, by highlighting the inter-group conflict between "natives" and "immigrants"? If this is the case, then the effects of social categorization (as described by Tajfel and Turner, among others) should be considered in the chapter on "theory and hypotheses," as well as in the discussion. In my opinion, the salience of these social categories and the subsequent conflict between the two groups is the necessary precondition for the occurrence of the Stereotype Lift effect, particularly in the authors' study.

We are happy to hear that you consider our work original, rigorous, and important. Pertaining to your first major comment, thank you for bringing your concern regarding how natives are impacted to our attention. We argue that, in regions with higher far-right political support, immigrants are negatively stereotyped (i.e., devalued) and that natives are made aware of this through public discourse but also symbolic cues such political signs and apparel. As a result of favorable downward negative social comparisons with immigrants, natives in these regions experience more self-efficacy and self-worth, and less self-doubt or anxiety. These effects, in turn, translate into increased performance because feelings of increased self-confidence will decrease self-monitoring, the direction of attention to automatic tasks (e.g., dribbling a basketball) negatively impacting immigrant players in regions higher in far-right political support. This mechanism, via the positive outcomes of favorable downward social comparisons, has been proposed and tested in numerous studies on stereotype lift (Chalabaev, Stone, Sarrazin, & Croizet, 2008; Estes & Felker, 2012; Laurin, 2013; Walton & Cohen, 2003).

We agree that the salience of social categorization into in- and out-groups (i.e., natives and immigrants) influence individuals work-related performance outcomes and, for this reason, we selected regional far-right political support as our independent variable given far-right political groups’ heavy focus, and public rhetoric, on the topic of immigration. Far-right political parties thereby make the social identities of individuals (both immigrants and natives) salient. Furthermore, and though we also agree that perceived inter-group conflict stemming from social categorization has the potential to influence natives’ stereotype lift effects (e.g., Chatard et al., 2008), we do not discuss this in depth in our paper because our study context involves team members whose individual success is intimately entwined with their team’s success. For this reason, we limit our theorizing to natives’ awareness of immigrants’ devaluation in the local region without extending it to include natives’ perceptions of conflict (e.g., competition) with, or negative attitudes towards (e.g., prejudice), their fellow immigrant team members. Future studies could however expand on our model and provide more nuance regarding who is most susceptible to stereotype/lift effects by exploring how inter-group conflict and related constructs such as prejudice affect immigrant and natives’ work-related performance. We now discuss this in the Limitations and future directions section of our revised manuscript (p. 23).

Another theoretical issue that I believe requires further nuance in the discussion is the significant difference between the work context of an NBA player (or other sports with a mass audience) and any other work organization. From a psychosocial perspective, the public exposure of these "workers" in the NBA is a variable that should be considered more rigorously in the interpretation of the results. The authors should elaborate more on this aspect, as it does not invalidate their main findings but would add more value to the article. "The public exposure of these "workers" in the NBA, which distinguishes them from general workers, should be considered more carefully in the limitations section or as a topic for future work."

Thank you for your guidance on how to emphasize the uniqueness of the worker sample that we draw on. We wholeheartedly agree that the degree of exposure that NBA (or other sports) players have is likely a relevant factor in predicting their work-related and performance-related outcomes. Based on your comment, we now discuss in our limitations and future directions section (pp. 22-23) how exposure to an audience may moderate the relationships we report in our study. Furthermore, we also now note how studies on exposure as a predictor variable (i.e., moderator) could extend, or offer limiting conditions to, our theoretical model.

We hope that we could fully address your concerns with our revised manuscript. Should additional clarification be required, or additional concerns arise, we look forward to addressing them in another revision round.

References

Chalabaev, A., Stone, J., Sarrazin, P., & Croizet, J. (2008). Investigating physiological and self-reported mediators of stereotype lift effects on a motor task. *Basic and Applied Social Psychology*, *30*(1), 18–26. doi: 10.1080/01973530701665256

Chatard, A., Selimbegović, L., Konan, P., & Mugny, G. (2008). Performance boosts in the classroom: Stereotype endorsement and prejudice moderate stereotype lift. *Journal of Experimental Social Psychology*, *44*(5), 1421-1424.

Estes, Z., Felker, S. (2012). Confidence mediates the sex difference in mental rotation performance. *Archives of Sexual Behavior,* *41*, 557–570. doi:10.1007/s10508-011-9875-5

Laurin, R. (2013). Stereotype threat and lift effects in motor task performance: The mediating role of somatic and cognitive anxiety, *The Journal of Social Psychology*, *153*(6), 687–699, doi:10.1080/00224545.2013.821098

Walton, G., & Cohen, G. (2003). Stereotype lift. *Journal of Experimental Social Psychology*, *39*(5), 456–467. doi: 10.1016/s0022-1031(03)00019-2
