# Supplementary material for: Political context and immigrants’ work-related performance errors: Insights from the National Basketball Association
Source: PLoS One. 2023 Nov 1;18(11):e0289019. doi: 10.1371/journal.pone.0289019 (PMC10619861; doi:10.1371/journal.pone.0289019)
Supplement: Attachment — Submitted filename: Response_to_Reviewers.docx [file pone.0289019.s002.docx]

Dear Dr. Vousden and Dr. Zhang,

Thank you for giving us another opportunity to revise our manuscript and bring it in line with PLOS ONE reporting standards. Below we address your comments, with each of our responses presented in blue.

The submitted manuscript is observational in nature. As such, it is not appropriate to make draw conclusions related to causation. The following fragments must be revised to indicate that an association between the variables of note was reported, but must not imply that the results demonstrate causation or direction of a given effect. You may speculate on the potential for causation in your manuscript, but not suggest your results are sufficient to demonstrate this.

We understand the importance of emphasizing that our findings are correlational in nature and appreciate your detailed insight on which parts of the text require changing and how they can be effectively changed.

Line number below refer to the clean version of the manuscript in Editorial Manager.

Line 80-82: “can be predisposed” should be revised (e.g. with “show increased performance errors”)

This section of text has been replaced with the text you proposed.

Line 88-90: This statement implies causation and must be revised.

This has been revised.

Line 96-99: This statement implies causation and must be revised.

This has been revised.

Line 336-339: This statement implies causation and must be revised.

This has been revised.

Line 353-356: Whilst most of the text in lines 353-355 is acceptable, “jeopardizing” implies causation and should be revised.

This has been revised.

Line 372-374: This statement implies causation and must be revised.

This has been revised.

Line 374:377: This statement implies causation and must be revised.

This has been revised.

Limitations section: Please extend the discussion beginning on line 442 to include how the observational nature of the data prevents conclusions related to causation being made.

As you request, we now mention that the observational nature of our data preclude causal claims and that future studies are needed to establish causality.

Line 460-462: the text beginning ‘thus’ should be toned down. Whilst you may speculate on the causes of the patterns observed here, this sentence overstates your conclusions. It would be acceptable to indicate as follows:
“Specifically, we demonstrate the positive link between regional far-right political support and performance errors in immigrants (regardless of their skin color). This raises the possibility that the external political environment can result in negative consequence for immigrants and the organizations for whom they work.”

This has been revised. Thank you for providing an adequate alternative to what we originally wrote, this has eased the revision process.

In addition to the above, please also amend your Methods section to provide further detail on how immigrant status was determined. The specific data sources used to determine this should be required. In addition, it is stated that the approach was “similar to other research [29]”. Please supplement this reference with references to original research studies using the same approach as described here.

As you request, we now specify where exactly the information on immigrant status came from and cite previous research studies operationalizing immigrant status based on study participants’ country of birth.

We hope you find the changes in this revision satisfactory but stand ready to make additional alterations if necessary.
